# Dissipative Continuous Spontaneous Localization (CSL) model

**DOI:** 10.1038/srep12518

**Published:** 2015-08-05

**Authors:** Andrea Smirne, Angelo Bassi

**Affiliations:** 1 Dipartimento di Fisica, Università degli Studi di Trieste, Strada Costiera 11, I-34151 Trieste, Italy; 2 Istituto Nazionale di Fisica Nucleare, Sezione di Trieste, Via Valerio 2, I-34127 Trieste, Italy

## Abstract

Collapse models explain the absence of quantum superpositions at the macroscopic scale, while giving practically the same predictions as quantum mechanics for microscopic systems. The Continuous Spontaneous Localization (CSL) model is the most refined and studied among collapse models. A well-known problem of this model, and of similar ones, is the steady and unlimited increase of the energy induced by the collapse noise. Here we present the dissipative version of the CSL model, which guarantees a finite energy during the entire system’s evolution, thus making a crucial step toward a realistic energy-conserving collapse model. This is achieved by introducing a non-linear stochastic modification of the Schrödinger equation, which represents the action of a dissipative finite-temperature collapse noise. The possibility to introduce dissipation within collapse models in a consistent way will have relevant impact on the experimental investigations of the CSL model, and therefore also on the testability of the quantum superposition principle.

The superposition principle lies at the core of quantum mechanics. The last years have experienced a huge progress in the theoretical and experimental investigation aimed at preparing and observing quantum superpositions of large systems^1^,^2^,^3^,^4^,^5^,^6^,^7^. Such a progress promises a crucial insight into a question which was born with quantum mechanics itself^8^: Can quantum mechanics be applied at all scales, including the macroscopic ones, or is there an intrinsic limit, above which its description of reality is not appropriate? Collapse models^9^,^10^,^11^,^12^,^13^ show explicitly how the second point of view can be assumed without the need to introduce an ad-hoc separation between the microscopic and the macroscopic world within the theory^14^. Through a non-linear stochastic modification of the Schrödinger equation, collapse models predict a behavior of microscopic systems which almost strictly follows that of standard quantum mechanics, while preventing macroscopic systems from being in a superposition of macroscopically distinct positions.

The continuous spontaneous localization (CSL) model^11^ is the most refined collapse model, as it also applies to identical particles. The mass density of a quantum system is coupled with a white-noise field, which can be interpreted as a classical random field filling space^13^. Different speculations on the origin of the noise field have been developed, tracing it back, e.g., to gravity^15^ or to cosmological particles^16^. However, the full characterization of such a noise calls for a new fundamental theory, which departs from quantum mechanics and can explain the classical nature of the noise, as well as its non-hermitian and non-linear coupling with matter^13^,^17^. In this respect, the CSL model, like every collapse model, should be seen as a phenomenological model expressing the influence of the noise field in an effective way.

The localization of the wavefunction of macroscopic objects, along with the resulting destruction of quantum superpositions, is not the only distinctive feature of the CSL model with respect to the usual Schrödinger evolution. The action of the noise induces a steady increase of the mean kinetic energy, which diverges on the long time scale^11^, thus manifestly leading to a violation of the principle of energy conservation. Despite the smallness of the increase rate, the comparison of the predictions on the secular energy with cosmological data provides some of the strongest experimental bounds on the two intrinsic parameters of the model^18^,^19^. In particular, the spontaneous heating of the intergalactic medium which would be induced by the stochastic noise sets *λ* ~ 10^−9^ s^−1^ as an upper bound to the localization rate *λ*; this value coincides with the proposal by Adler based on the analysis of latent image formation in photography^18^.

As one may easily imagine, a significant and long-time debated^20^,^21^,^22^,^23^,^24^ issue is whether the divergence of the energy in collapse models can be avoided, thus pointing to a reestablishment of the energy conservation principle, while preserving the specific features any collapse model must have. In^25^ we showed how this can be attained for the Ghirardi-Rimini-Weber (GRW)^9^ model. In this work, we move a step forward and we introduce the dissipative CSL model, thus getting a collapse model which both applies to (non-relativistic) identical particles and keep the energy finite on the whole time scale. We modify the defining stochastic differential equation via the introduction of new operators, which depend on the momentum of the system. This determines the occurrence of dissipation^26^,^27^,^28^, thus leading to the relaxation of the energy to a finite asymptotic value. The latter can be associated with a finite temperature of the noise field. Remarkably, such a temperature can take on small values (of the order of 1 K) while the effectiveness of the model is maintained. Therefore, contrary to a common misconception, the steady increase of the energy is not an unavoidable trait of collapse models inducing localization in space: in our dissipative model there is a continuous localization of the wavefunction, while the mean energy of the system will typically decrease.

Using the language of non-relativistic quantum field theory, the CSL model is formulated in terms of a stochastic differential equation in the Fock space associated with the system^11^. Given different types of particles, where the type *j* has mass *m*_*j*_, the mass-proportional CSL model^29^ is defined by


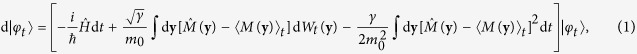


where 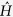 is the standard quantum Hamiltonian, 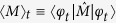, *m*_0_ is a reference mass (usually the mass of a nucleon) and 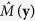 is a smeared mass density operator:


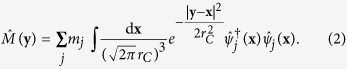


Here, 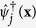 and 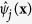 are, respectively, the creation and the annihilation operator of a particle of type *j* in the point **x**, while *W*_*t*_(**y**) is an ensemble of independent Wiener processes, one for each point in space. The model is characterized by two parameters: *γ*, which sets the strength of the collapse process, and *r*_*C*_, which determines the threshold above which spatial superpositions are suppressed. The choice of the numerical values for these parameters is of course ultimately dictated by the agreement with experimental data; the originally proposed values are^11^ *r*_*C*_ = 10^−7^ m and *γ* = 10^−30^ cm^3^ s^−1^.

The mass density operators 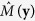 in Eq. (1) induce a collapse of the wavefunction |*φ*_*t*_〉 around the common eigenvectors of the position operators of the particles composing the system^11^. Hence, the asymptotic wavefunction is sharply localized around definite positions, excluding possible spatial superpositions. The collapse rate for a microscopic system is given by 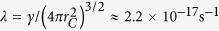. Such a small value guarantees that the spatial localization due to the noise field can be safely neglected if a microscopic system is taken into account. Now instead, consider a macroscopic rigid body in a superposition of two states distant more than *r*_*C*_. Its center of mass collapses with an effective rate^18^,^30^


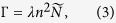


where *n* is the number of constituents of the body contained in a volume 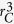 and 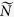 denotes how many such volumes are held in the macroscopic body. This relation clearly shows the amplification mechanism, which is at the basis of every collapse model. The localization induced by the noise field grows with the size of the system, so that the center of mass of any macroscopic object behaves, for all practical purposes, according to classical mechanics. The peculiar property of the CSL model is the quadratic dependence of the rate Γ on the number of constituents, which is a direct consequence of the action of the noise field on identical particles^13^. The main features of the CSL model are summarized in Fig. 1, where we represent the time evolution of the position probability distribution of one particle, which is initially in a superposition of two gaussian states. The wavefunction is subject continuously to the action of the noise, which suppresses the superposition between the two gaussians, leading to a gaussian state localized around one of the two initial peaks, in a time scale fixed by the collapse rate, see Fig. 1(left, center). The diffusive nature of the dynamics in the CSL model is clearly illustrated by the time-evolution of the position variance, see Fig. 1(right).

As already mentioned, a relevant drawback of the original CSL model, as well as of most collapse models, is that the average kinetic energy of the quantum system diverges on the long time scale^9^,^11^,^20^. The model predicts that the energy of a particle with mass *m* increases linearly in time with a rate 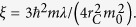 As will become clear by the following analysis, the reason for such an energy increase is precisely due to the absence of dissipation within the model. The noise acts like an infinite temperature background, steadily increasing the energy of the system.

## Results

### Dissipative extension of the CSL model

Now that we have clarified the problem of the CSL model we want to work out, as well as the features that must be preserved, we are in the position to formulate a new, dissipative CSL model. As for the original model, the most compact way to do so is to define a proper stochastic differential equation. Specifically, we consider the following non-linear stochastic differential equation:


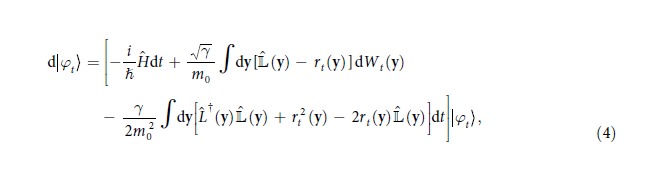


with 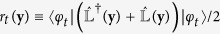 and


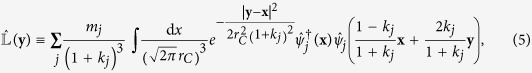


where *k*_*j*_ ≡ *ħ*/(2*m*_*j*_*v*_*η*_*r*_*C*_). A linear stochastic differential equation can be associated with the model, as well, see Supplementary Information. The inclusion of dissipation calls for the introduction of a new parameter, *v*_*η*_, with the dimension of a velocity. Crucially, this parameter is related to the temperature of the noise field, as it will be shown later (see Eq. (8)), where the numerical choice of *v*_*η*_ will be also discussed. The structure of the stochastic differential equation (4) generalizes that of Eq. (1) to the case of non self-adjoint operators^31^,^32^. Indeed, for *v*_*η*_ → ∞, so that *k*_*j*_ → 0, one recovers the original CSL model.

The physical meaning of the operator 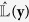 in Eq. (5) is better understood by taking into account also its momentum representation. One has


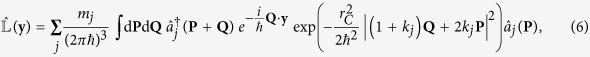


where 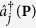 and 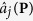 are, respectively, the creation and annihilation operator of a particle of the type *j* with momentum **P**. By Eqs (5) and (6), we see that the action of the collapse noise can be compared to that of an external potential which depends not only on the position, but also on the momentum of the system, thus inducing dissipation. In particular, since the exchanged momentum *Q*_*i*_ in the spatial direction *i* = *x*, *y*, *z* has a gaussian distribution peaked around −2*P*_*i*_*k*_*j*_/(1 + *k*_*j*_), the action of the noise will suppress high momenta, so that the mean kinetic energy of the system, as well as the mean momentum, is subject to relaxation.

Before showing that, we would like to remark that the collapse noise, contrary to any external field, has an anti-hermitian coupling with matter, which is necessary in order to induce localization. In addition, the introduction of dissipation also leads to an hermitian contribution to the coupling, see Supplementary Information for details.

### Energy relaxation and noise temperature

For the sake of simplicity, we deal with the average dynamics experienced by a single particle of mass *m* under the action of a noise field as in Eq. (4). More details about the system’s master equation and the calculations needed to derive the following results are contained in the Methods section.

We denote as *H*(*t*) the stochastic average of the mean kinetic energy performed over the different trajectories of the model, i.e. 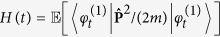, where 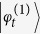 solves the stochastic differential equation (4) restricted to the one-particle sector of the Fock space and 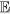 is the stochastic average with respect to the reference probability 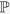. The master equation for the one-particle average state 
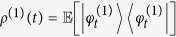
 directly provides us with


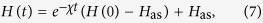


with relaxation rate 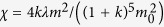, where *k* = *ħ*/(2*mv*_*η*_*r*_*C*_), and asymptotic kinetic energy 
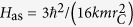
. As expected, now we do have dissipation. The mean energy of the system can decrease as a consequence of the action of the noise. Moreover, even if the energy grows, there is an upper threshold value above which it cannot increase. The long-time energy divergence is now avoided; note that since the average mean kinetic energy is finite, the mean kinetic energy is almost surely finite on each trajectory. This is precisely the result we wanted and the most natural way to interpret it is to say that the collapse noise has a finite temperature toward which the system thermalizes^23^. Explicitly, *H*_as_ corresponds to a noise temperature


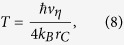


where we used *k* = *ħ*/(2*mv*_*η*_*r*_*C*_) and *k*_*B*_ is the Boltzmann constant. The original CSL model is recovered in the limit *T* → ∞: in that case the noise acts like an infinite temperature background, which explains the energy divergence.

The temperature of the noise in Eq. (8) does not depend on the mass of the system, which is a very important feature of our model. In addition, the state of the system actually equilibrates to the canonical Gibbs state. These hallmarks of the evolution induced by Eq. (4) depend substantially on the choice of the operators 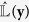 in Eq. (5). It is an open question to identify the entire class of operators satisfying these natural requests. In the Supplementary Information, we take into account a physically motivated alternative to the choice made in Eq. (5), showing how the relaxation dynamics of the resulting collapse model is very similar to that presented here and, in particular, the noise temperature is still given by Eq. (8). The exponential relaxation of the energy *H*(*t*) in Eq. (7) is the same as that in the dissipative GRW model^25^. This is not surprising, since, as for the case without dissipation, the extended GRW and CSL models share the same one-particle master equation.

If we think that the collapse model fixed by Eq. (4) describes the action of a real physical field filling space, it is now clear how the principle of energy conservation can be reestablished. The energy gained or lost by the system can be ascribed to an energy exchange with the noise field, as the latter can be influenced back by the presence of the system. An explicit characterization of this process requires an underlying theory, which has to guarantee the classical nature of the noise field, with its own (non-quantum) equations of motion, in order to provide a proper objective collapse of the wavefunction^12^,^13^,^17^. In addition, one can already say that a collapse noise with typical cosmological features would correspond to a low-temperature noise^13^,^33^, at most of the order of few Kelvins. By Eq. (8), we see that the noise temperature *T* is in one-to-one correspondence with the new parameter *v*_*η*_. For instance, *v*_*η*_ = 10^5^ m/s (i.e. *k* ≈ 3 × 10^−6^ for a nucleon) gives *T* ≈ 1 *K*. Hence, more than the specific value of the noise temperature, the important thing is that even in the presence of a low-temperature noise the resulting collapse model is effective, as shown in the next paragraph. It is worth noting that it is not always possible to properly modify a given collapse model to include dissipation via the action of a low-temperature noise^34^.

In our model, every system with a temperature higher than about 1 K is cooled by the action of the collapse noise. Thus, we are led to reject the bounds on the collapse rate *λ* relying on a balance between the system’s heating due to the action of the noise in the original CSL model and the cooling due to, e.g., the Universe expansion or the energy radiation. This is the case for the heating of the protons constituting the intergalactic medium (IGM) or for the energy accumulation in interstellar dust grains^18^. Note how, in particular, the heating of the IGM provides the second strongest bound to date on the localization rate *λ*^19^. Even more, we expect that cosmological data will put strong bounds on the dissipation parameter *k* (equivalently, on *v*_*η*_). The modified long-time behavior of the energy predicted by our model will have to be compared with the constraints coming from such data. Some preliminary results have been obtained for the non-dissipative CSL model^18^,^35^. Dissipative effects are expected to play an important role also in the experimental investigation of collapse models via optomechanical systems^36^,^37^, where proper signatures could be visible in the density noise spectrum of the mechanical oscillator, or via the spontaneous photon emission from electrically charged particles^38^,^39^, as the latter is registered over a period of several years. In both situations, we expect that dedicated experiments should allow to restrict the possible values of *k*; of course, also in relation with the other parameters of the model.

### Macroscopic objects: localization and amplification mechanism

As recalled in the Introduction, any proper collapse model is characterized by the amplification mechanism. The localizing action of the collapse noise has to increase with size of the system, which guarantees a consistent description of microscopic and macroscopic systems within a unique theoretical framework. Here, we show that the amplification mechanism holds in our extended model, at least as long as one deals with a macroscopic rigid body. The description of more complex systems, where the internal dynamics affects the evolution of the center of mass, calls for a more detailed specification of the system’s evolution^25^. We stress that the following considerations are valid also in the case of a low temperature noise. As anticipated, even for a noise temperature *T* ≈ 1 *K* we have effective localization and amplification mechanisms, so that the noise actually induces a classical behavior of the center of mass of macroscopic objects.

Consider a macroscopic object made up of *N* particles of equal mass *m*. We deal with a rigid body, which allows us to decouple the evolution of the center of mass from that of the relative coordinates^25^. Let 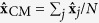 be the position operator of the center of mass, while the relative coordinates 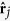, *j* = 1,…, *N*−1, are fixed by 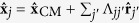, for a suitable matrix with elements Λ_*jj*′_. We neglect the possible rotations of the system: this greatly simplifies the description, without affecting the physical meaning of the results^11^. In addition, consider a total Hamiltonian 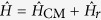, given by the sum of two terms associated with, respectively, the center of mass and the relative degrees of freedom. It is easy to see that the state of the center of mass 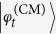 satisfies a stochastic differential equation with the same form as Eq. (4), where 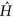 is replaced by 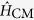 and 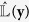 is replaced by


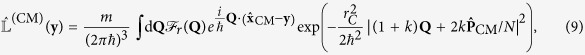



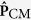
 being the center-of-mass momentum operator and we introduced the function 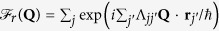, where **r**_*j*_ is the fixed *j*-th relative coordinate of the rigid body. The factor 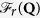 conveys the influence of the internal structure on the evolution of the center of mass and it is due to the indistinguishability of particles: it is also present in the original CSL model^11^, but not in the GRW model^9^,^25^.

**Figure 1:**
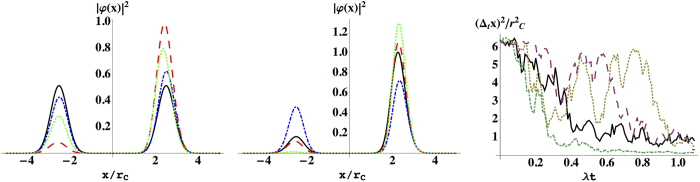
(Left, center) Evolution of the position probability distribution 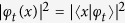 in the CSL model in one dimension, for one nucleon initially in a balanced superposition of two gaussian states with equal variance *σ*^2^ and centered, respectively, in *α* and −*α*. The probability distribution is plotted for a single realization of the random noise and at times *λt* = 0 (black solid line), *λt* = 0.1 (blue dot-dashed line), *λt* = 0.3 (red dashed line) and *λt* = 0.4 (green dotted line), left, and *λt* = 0.5 (black solid line), *λt* = 0.6 (blue dot-dashed line), *λt* = 0.8 (red dashed line) and *λt* = 0.9 (green dotted line), (center); *σ*/*r*_*C*_ = 0.55 and *α*/*r*_*C*_ = 2.5. (Right) Time evolution of the position variance, 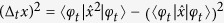, for different realizations of the noise field. We have applied the Euler-Maruyama method^47^,^48^ to Eq. (1), for 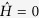 and time step *λ*Δ*t* = 0.01. As discussed in the text, see also Supplementary Information for more details, the rate *λ* has to be replaced by the rate Γ defined in Eq. (3) if a macroscopic object is taken into account, in accordance with the amplification mechanism.

Let us take into account the continuum limit 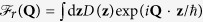, where *D*(**z**) is the density of particles and assume that this macroscopic density does not vary significantly on the length-scale fixed by *r*_*C*_. In the Supplementary Information, we show that the effects on the localization process due to the presence of the momentum operator in 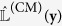 can be then safely neglected, so that the convergence toward well localized states is still guaranteed. The localization of the wavefunction, as, e.g., represented in Fig. 1, is basically not modified by the introduction of dissipation in the model. Moreover, the amplification mechanism can be characterized through Eq. (3). The localization rate is vey small for microscopic systems, while increasing with the size of the system proportionally to the square of the number of particles, which is a direct signature of the action of the noise on indistinguishable particles.

The comparison between the dissipation rate *χ* and the localization rate Γ, see Eq. (3), shows how the two phenomena occur on different time scales: while the center of mass of a macroscopic system will be quickly localized by the action of the noise, dissipation can possibly play a role on the system’s evolution only on the long time scale. This explains how the introduction of momentum-dependent localization operators can leave the localization and amplification mechanisms unchanged, while it modifies significantly the long-time behavior of the system’s energy. Explicitly, let us take into account the evolution of the center-of-mass energy of a macroscopic rigid body with *N* nucleons, 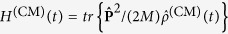, where *M* = *Nm*_0_ is the total mass. At first order in *k*, the center-of-mass master equation leads to an exponential relaxation of the energy with rate


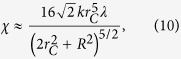


where we considered a spherical object with radius *R* and constant density and we used 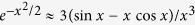. Evaluating the localization rate Γ via Eq. (3), one gets that the ratio between the two rates, in the case *R* ≫ *r*_*C*_, is


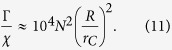


If we consider a reference density *D* = 5 g cm^−3^, one has *N* ≈ 10^25^(*R*[c*m*])^3^, and, setting a radius *R* = 1 mm, we have *N* ≈ 10^22^. In this case, the localization rate is Γ = 10^14^ s^−1^, while the dissipation rate is *χ* = 10^−41^ s^−1^: the noise localizes the center of mass of the macroscopic body on very short time scales, while the influence of dissipation can be safely neglected during the whole evolution of the macroscopic system. Similarly, if *R* = *r*_*C*_ = 10^−5^ cm, implying *N* ≈ 10^10^, we get *χ* = 10^−22^ s^−1^, while in this case Γ ≈ 10^2^ s^−1^. Moreover, one could wonder how this analysis changes if we choose a different one-particle localization rate *λ*. For the value proposed by Adler^18^, *λ* = 10^−9^ s^−1^, we have that dissipation can still be neglected for *R* = 1 mm, where *χ* = 10^−33^ s^−1^ (and Γ = 10^22^ s^−1^). Instead, for *R* = *r*_*C*_ = 10^−5^ cm, we end up with *χ* = 10^−14^ s^−1^, so that dissipation will play a role on the secular evolution of the system. However, also in this case the effect of dissipation on the localization of the wavefunction is completely negligible. Localization occurs on a much shorter time scale than dissipation, Γ = 10^10^ s^−1^, and then the influence of the dissipative terms can be neglected to study localization, even if it can subsequently play a role in the long-time behavior of the system.

## Discussion

The main purpose of collapse models is to provide a unified framework for the description of microscopic and macroscopic systems, thus avoiding an ad-hoc dividing line within the theory, as well as yielding a dynamical explanation for the collapse of the wavefunction. The results of this paper point out that this program can be followed by taking into account basic physically-motivated demands.

We have included dissipation in the CSL model, which is up to now the most refined collapse model. This allowed us to remove the divergence of the energy on the long time scale affecting the original CSL model. This divergence traces back to an infinite temperature of the collapse noise, which is of course an unrealistic feature of the model. The inclusion of dissipation brings along a new parameter, which is strictly related with the finite temperature of the noise. Significantly, even in the presence of a low-temperature noise the localization and the amplification mechanism are effective, so that the unified description of microscopic and macroscopic systems is still guaranteed.

A realistic description of the wavefunction collapse can be further developed, for example by also including a non-white noise^16^,^40^ within the model. Nevertheless, one should keep in mind that the specific features of the collapse noise can be fixed only through a first-principle underlying theory, which can clarify the physical origin of the noise^13^,^41^. The development of such an underlying theory is one of the main goals of the research on collapse models and, more in general, on the theories going beyond quantum mechanics.

## Methods

Here, we show explicitly how the stochastic differential equation (4) implies that the statistical operator satisfies a Lindblad master equation. After presenting the equation in a second-quantization formalism, we describe the corresponding operators in the case of a fixed number of particles. In particular, by focusing on the one-particle case, we derive Eq. (7).

The stochastic differential equation fully fixes the collapse model we are defining here. However, one is often interested in the predictions of the model related with the statistical mean of some physical quantity,


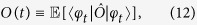


where, as usual, |*φ*_*t*_〉 is the stochastic state of the system satisfying Eq. (4). For this reason, it can be convenient to deal directly with the evolution of the average state


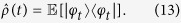


In particular, by using the Itô calculus, it is easy to see that Eq. (4) implies that 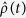 satisfies the following master equation:


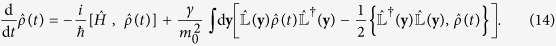


This is a Lindblad master equation^42^,^43^,^44^, indicating that we are in the presence of a Markovian dynamics. The Lindblad operators are the same operators as those appearing in the stochastic differential equation defining the model, see Eq. (5) or (6).

It is useful also to consider the explicit expressions of the Lindblad operators 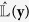 when we restrict to a sector of the Fock space with a fixed number of particles. Let us assume for simplicity that we have *N* particles of the same type and mass *m*. The corresponding restriction of 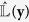 reads


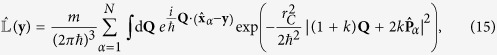


where 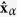 and 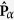 are, respectively, the position and momentum operator of the *α*-th particle and *k* is the constant


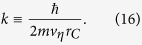


In fact, consider the Hilbert space 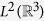 and the corresponding Fock space 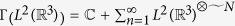, where 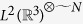 denotes the symmetric or antisymmetric part of the tensor product 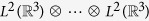, *N* times. Now consider the operator on 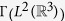 given by^45^


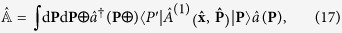


where 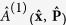 is a single-particle operator on 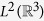, with 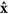 and 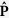, respectively, position and momentum operators on 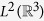. The restriction of 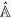 on the *N*-particle sector of the Fock space reads


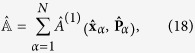



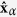
 and 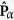 being the position and momentum operator of the *α*-th particle. The relation between Eq. (17) and Eq. (18) is indeed the same as that between Eq. (6) and Eq. (15).

If we further restrict to the case of a single free particle with mass *m*, we end up with the following master equation for the one-particle average state 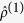



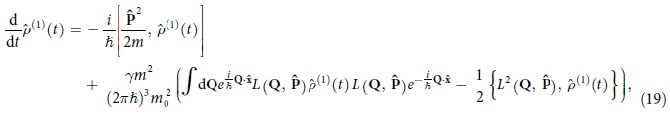


with


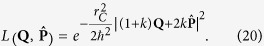


Apart from a different rate, this master equation precisely corresponds to that of the dissipative GRW model recently introduced in^25^, where more details about such a master equation can be found.

Using Eq. (19) we can directly compute the evolution equation of the mean kinetic energy 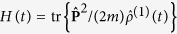; by exploiting the translation covariance of the master equation^28^,^46^ one easily gets


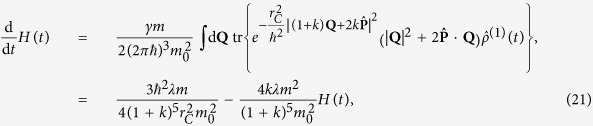


whose solution is given by Eq. (7); recall that *λ* has been defined as


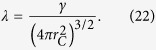


In addition, let us note that the inclusion of dissipation in the CSL model preserves the invariance under translations of the system’s evolution, but it breaks the invariance under boosts, as directly seen by the master equation (19)^46^. Nevertheless, the characterization of the overall dynamics by means of a proper first-principle underlying theory, which involves both the sources of the collapse noise and the quantum systems affected by it, should allow to recover a fully covariant description.

## Additional Information

**How to cite this article**: Smirne, A. and Bassi, A. Dissipative Continuous Spontaneous Localization (CSL) model. *Sci. Rep.* **5**, 12518; doi: 10.1038/srep12518 (2015).

## Supplementary Material

Supplementary Information
