# Supplementary material for: Dissipative Continuous Spontaneous Localization (CSL) model
Source: Sci Rep. 2015 Aug 5;5:12518. doi: 10.1038/srep12518 (PMC4525142; doi:10.1038/srep12518)
Supplement: Supplementary Information [file srep12518-s1.pdf]

Dissipative Continuous Spontaneous Localization (CSL) model: Supplementary Information

Andrea Smirne[1, 2, *] and Angelo Bassi[1, 2, †]

[1] *Dipartimento di Fisica, Università degli Studi di Trieste, Strada Costiera 11, I-34151 Trieste, Italy*
[2] *Istituto Nazionale di Fisica Nucleare, Sezione di Trieste, Via Valerio 2, I-34127 Trieste, Italy*

I. LINEAR STOCHASTIC DIFFERENTIAL EQUATION

Here, we briefly sketch the standard procedure which provides the linear stochastic differential equation associated with the non-linear one in the form given by Eq.(4) in the main text. For a complete treatment, the reader is referred to [1].

Hence, consider a non-linear stochastic differential equation as Eq.(4) in the main text. Recall that $W_t(\mathbf{x})$ denotes an ensemble of independent Wiener processes defined on a common probability space $(\Omega, \mathcal{F}, \mathbb{P})$. Let $B_t(\mathbf{x})$ be the ensemble of processes given by

$$B_t(\mathbf{x}) = W_t(\mathbf{x}) + 2\int_0^t \mathrm{d}s\, r_s(\mathbf{x}), \tag{1}$$

where $r_s(\mathbf{x})$ has been defined after Eq.(4) in the main text. Now, by means of the Girsanov theorem one can define a new probability $\mathbb{Q}$ on $(\Omega, \mathcal{F})$ such that $B_t(\mathbf{x})$ is an ensemble of Wiener processes under $\mathbb{Q}$ [1]. In addition, one can define a random vector $|\psi_t\rangle$ such that

$$|\varphi_t\rangle = \frac{|\psi_t\rangle}{\|\psi_t\|}, \tag{2}$$

where $|\varphi_t\rangle$ satisfies Eq.(4) of the main text, while $|\psi_t\rangle$ satisfies [1]

$$\mathrm{d}|\psi_t\rangle = \left[-\frac{i}{\hbar}\hat{H}\mathrm{d}t - \frac{\gamma}{2m_0^2}\int \mathrm{d}\mathbf{y}\hat{\mathbb{L}}^\dagger(\mathbf{y})\hat{\mathbb{L}}(\mathbf{y})\mathrm{d}t + \frac{\sqrt{\gamma}}{m_0}\int \mathrm{d}\mathbf{y}\hat{\mathbb{L}}(\mathbf{y})\mathrm{d}B_t(\mathbf{y})\right]|\psi_t\rangle. \tag{3}$$

This is the linear stochastic differential equation associated with the dissipative CSL model.

II. HERMITIAN AND ANTI-HERMITIAN COUPLING OF THE NOISE FIELD

In this section, we show that the introduction of dissipation within the CSL model leads to an hermitian contribution to the coupling of the collapse noise with quantum matter, in addition to a contribution in the usual (for collapse models) anti-hermitian form [2, 3].

It is here convenient to use the Stratonovich formalism [4, 5] and to consider the decomposition of $\hat{\mathbb{L}}(\mathbf{y})$ into its hermitian and anti-hermitian part, $\hat{\mathbb{L}}(\mathbf{y}) = \hat{\mathbb{L}}^{(a)}(\mathbf{y}) + i\hat{\mathbb{L}}^{(b)}(\mathbf{y})$, with $\hat{\mathbb{L}}^{(a)}(\mathbf{y})$ and $\hat{\mathbb{L}}^{(b)}(\mathbf{y})$ self-adjoint operators. Thus, the non-linear stochastic equation given by Eq.(4) in the main text leads to

$$\frac{\mathrm{d}|\varphi_t\rangle}{\mathrm{d}t} = \left[-\frac{i}{\hbar}\left(\hat{H} - \frac{\sqrt{\gamma}\hbar}{m_0}\int \mathrm{d}\mathbf{y}\hat{\mathbb{L}}^{(b)}(\mathbf{y})w(\mathbf{y},t) + R\right) + \frac{\sqrt{\gamma}}{m_0}\int \mathrm{d}\mathbf{y}\hat{\mathbb{L}}^{(a)}(\mathbf{y})w(\mathbf{y},t) + S\right]|\varphi_t\rangle, \tag{4}$$

where $w(\mathbf{x},t)$ is the white-noise field, which can be formally written as $w(\mathbf{x},t) = \mathrm{d}W_t(\mathbf{x})/\mathrm{d}t$ and satisfies the relations

$$\mathbb{E}[w(\mathbf{x},t)] = 0 \qquad \mathbb{E}[w(\mathbf{x},t)w(\mathbf{y},t')] = \delta(\mathbf{x}-\mathbf{y})\delta(t-t'), \tag{5}$$

$\mathbb{E}$ being the stochastic average under the reference probability $\mathbb{P}$. Moreover, R is an hermitian contribution coming from the passage to the Stratonovich formalism and reads

$$R = -\frac{\gamma\hbar}{m_0^2}\int \mathrm{d}\mathbf{y}\left(\hat{\mathbb{L}}^{(b)}(\mathbf{y})\hat{\mathbb{L}}^{(a)}(\mathbf{y}) - 2\hat{\mathbb{L}}^{(b)}(\mathbf{y})\langle\hat{\mathbb{L}}^{(a)}(\mathbf{y})\rangle_t - \langle\hat{\mathbb{L}}^{(b)}(\mathbf{y})\hat{\mathbb{L}}^{(a)}(\mathbf{y})\rangle_t\right). \tag{6}$$

---

[*] andrea.smirne@ts.infn.it
[†] bassi@ts.infn.it

On the other hand, S includes the non-linear contributions preserving the norm of the state vector and is given by

$$S = -\frac{\gamma}{m_0^2}\int \mathrm{d}\mathbf{y}\left[\left(\hat{\mathbb{L}}^{(a)}(\mathbf{y}) - \langle\hat{\mathbb{L}}^{(a)}(\mathbf{y})\rangle_t\right)^2 + (\langle\hat{\mathbb{L}}^{(a)}(\mathbf{y})\rangle_t)^2 - \langle(\hat{\mathbb{L}}^{(a)}(\mathbf{y}))^2\rangle_t\right] - \frac{\sqrt{\gamma}}{m_0}\int \mathrm{d}\mathbf{y}\langle\hat{\mathbb{L}}^{(a)}(\mathbf{y})\rangle_t w(\mathbf{y},t), \tag{7}$$

where $\langle A\rangle_t \equiv \langle\varphi_t|\hat{A}|\varphi_t\rangle$; compare with Eq.(7.43) in [5].

Equation (4) describes the coupling between the classical field $w(\mathbf{x},t)$ and quantum matter. Now, since $\hat{\mathbb{L}}(\mathbf{y})$ is not a self-adjoint operator such a coupling has an hermitian, as well as an anti-hermitian contribution. Note that in the original CSL model the collapse noise is coupled with matter only via an anti-hermitian term [3]. To be explicit, Eq.(6) of the main text implies

$$\hat{\mathbb{L}}^{\dagger}(\mathbf{y}) = \sum_j \frac{m_j}{(2\pi\hbar)^3}\int \mathrm{d}\mathbf{P}\mathrm{d}\mathbf{Q}\,\hat{a}_j^{\dagger}(\mathbf{P}+\mathbf{Q})\,e^{-\frac{i}{\hbar}\mathbf{Q}\cdot\mathbf{y}}\exp\left(-\frac{r_C^2}{2\hbar^2}\left|(1-k_j)\mathbf{Q} - 2k_j\mathbf{P}\right|^2\right)\hat{a}_j(\mathbf{P}) \tag{8}$$

and thus

$$\begin{aligned}\hat{\mathbb{L}}^{(a)}(\mathbf{y}) = \sum_j \frac{m_j}{(2\pi\hbar)^3}\int \mathrm{d}\mathbf{P}\mathrm{d}\mathbf{Q}\,\hat{a}_j^{\dagger}(\mathbf{P}+\mathbf{Q})\hat{a}_j(\mathbf{P})\exp\left(-\frac{r_C^2}{2\hbar^2}\left(|\mathbf{Q}|^2 + k_j^2|\mathbf{Q}+2\mathbf{P}|^2\right)\right) \\ \times\cosh\left(\frac{k_j r_C^2}{\hbar^2}\mathbf{Q}\cdot(\mathbf{Q}+2\mathbf{P})\right)\end{aligned} \tag{9}$$

and

$$\begin{aligned}\hat{\mathbb{L}}^{(b)}(\mathbf{y}) = -\sum_j \frac{m_j}{(2\pi\hbar)^3}\int \mathrm{d}\mathbf{P}\mathrm{d}\mathbf{Q}\,\hat{a}_j^{\dagger}(\mathbf{P}+\mathbf{Q})\hat{a}_j(\mathbf{P})\exp\left(-\frac{r_C^2}{2\hbar^2}\left(|\mathbf{Q}|^2 + k_j^2|\mathbf{Q}+2\mathbf{P}|^2\right)\right) \\ \times\sinh\left(\frac{k_j r_C^2}{\hbar^2}\mathbf{Q}\cdot(\mathbf{Q}+2\mathbf{P})\right).\end{aligned} \tag{10}$$

Of course, $\hat{\mathbb{L}}^{(b)}(\mathbf{y}) = 0$ for $k_j = 0$.

III. A DIFFERENT CHOICE OF THE COLLAPSE OPERATORS $\hat{\mathbb{L}}(\mathbf{y})$

In this section, we discuss an alternative and physically motivated choice for the operators $\hat{\mathbb{L}}(\mathbf{y})$, which differs from that made in Eq.(5) (or, equivalently, in Eq.(6)) of the main text. We will see how this different choice does not affect significantly the features of the relaxation dynamics we are interested in. We also argue that in both cases the system's state equilibrates to the stationary solution in the Gibbs form.

Let us consider the following operators:

$$\hat{\mathbb{L}}(\mathbf{y}) = \sum_j \frac{m_j}{(2\pi\hbar)^3}\int \mathrm{d}\mathbf{P}\mathrm{d}\mathbf{Q}\,\hat{a}_j^{\dagger}(\mathbf{P}+\mathbf{Q})\,e^{-\frac{i}{\hbar}\mathbf{Q}\cdot\mathbf{y}}\exp\left(-\frac{r_C^2}{2\hbar^2}\left((1+k_j)Q + 2k_j\frac{\mathbf{P}\cdot\mathbf{Q}}{Q}\right)^2\right)\hat{a}_j(\mathbf{P}), \tag{11}$$

where Q denotes the modulus of $\mathbf{Q}$. Indeed, such operators still reduce to those of the original CSL model [6] in the limit $k_j \to 0$, i.e. $v_\eta \to \infty$. The difference with respect to the operators in Eq.(6) of the main text is that now the action of the noise no longer damps the momentum of the system in an isotropic way, but it tends to suppress the momentum of a particle in the state $|\mathbf{P}\rangle$ mainly in the direction $\mathbf{P}/P$. A significant motivation for collapse operators as in Eq.(11) would be their correspondence with the operators used to describe the collisional decoherence in the presence of dissipation. In particular, let us consider for simplicity the one-particle case. Then, the stochastic differential equation (4) of the main text, along with the operators in Eq.(11), provides us with a master equation for the average one-particle state $\hat{\rho}^{(1)}$ given by Eq.(19) of the main text, where $L(\mathbf{Q},\hat{\mathbf{P}})$ is now

$$L(\mathbf{Q},\hat{\mathbf{P}}) = e^{-\frac{r_C^2}{2\hbar^2}\left((1+k)Q + 2k\frac{\hat{\mathbf{P}}\cdot\mathbf{Q}}{Q}\right)^2}. \tag{12}$$

This master equation is the same as that describing the collisional dynamics of a test particle interacting with a low-density gas in the weak coupling regime [7], once we make a proper identification of the coefficients in the two models; see [8, 9] for more details.

By Eqs.(19) of the main text and (12), we directly have that the mean kinetic energy of the system evolves according to

$$\frac{\mathrm{d}}{\mathrm{d}t}H(t)=\frac{3\hbar^2\lambda m}{4(1+k)^5r_C^2m_0^2}-\frac{4(1-k)k\lambda m^2}{(1+k)^5m_0^2}H(t)-\frac{16k^3r_C^2M\lambda}{5\hbar^2(1+k)^5m_0^2}\langle P^4\rangle_t, \tag{13}$$

which involves the forth order momentum of the momentum operator and hence does not yield a closed equation for the mean kinetic energy. However, at first order in k this equation is exactly the same as Eq.(21) of the main text, so that one recovers an exponential relaxation to a finite asymptotic energy which corresponds to the noise temperature in Eq.(8) of the main text. Importantly, this expression of the noise temperature can be also proved exactly, by studying the relaxation to the equilibrium of the system's state. By direct substitution, one can easily see that the canonical Gibbs distribution

$$\varrho(\hat{\mathbf{P}})=\left(\frac{\beta}{2m\pi}\right)^{3/2}e^{-\frac{\beta|\hat{\mathbf{P}}|^2}{2m}} \tag{14}$$

is a stationary solution of Eq.(19) of the main text, with $L(\mathbf{Q},\hat{\mathbf{P}})$ as in Eq.(12), if $\beta=1/(k_BT)$ is the inverse temperature corresponding to Eq.(8) of the main text. Relying on the theorems by Spohn [10], we conclude that this stationary solution is unique and, for any initial condition, the state $\hat{\rho}^{(1)}(t)$ converges to it. Hence, the temperature in Eq.(8) of the main text is the temperature toward which the system thermalizes and is thus identified as the noise temperature. The theorems by Spohn apply when the set of Lindblad operators $V=\{L_j\}_{j=1\dots n}$ of a given Lindblad generator are such that only the multiples of the identity commute with all the elements of V and that if L_j is contained in V, then also $L_j^\dagger$ is so. Indeed, these two conditions are satisfied by set of the Lindblad operators in Eq.(12). To be precise, the theorems by Sphon hold in the finite dimensional case, so that, to put the above discussion on a firm mathematical ground, one should confine the whole system in a finite volume (which would also allow to take $\varrho(\hat{\mathbf{P}})$ as a proper quantum state) and include a cut-off on the momenta. In addition, once the uniqueness of the stationary state is guaranteed, the convergence to it for any initial condition can be alternatively proved by exploiting the contractivity of the relative entropy under completely positive and trace preserving maps [11].

The same results can be applied to our dissipative CSL model, i.e., if $L(\mathbf{Q},\hat{\mathbf{P}})$ is given by Eq.(20) of the main text. Beside providing a further proof of the Eq.(8) of the main text, this shows that our model predicts a convergence of the average one-particle state of the system to the stationary solution in the canonical form.

IV. LOCALIZATION OF THE CENTER OF MASS OF THE MACROSCOPIC SYSTEMS

Here, we provide more details about the estimate of the influence of dissipation on the localization of a macroscopic wavefunction, and we show that Eq.(3) of the main text still satisfactorily describes the amplification mechanism.

Let us consider the action of the operator $\mathbb{L}^{(\mathrm{CM})}(\mathbf{y})$, see Eq.(9) of the main text on a generic state $|\varphi^{(\mathrm{CM})}\rangle$ of the center of mass, such that $\langle\mathbf{x}|\varphi^{(\mathrm{CM})}\rangle=\varphi(\mathbf{x})$ and $\langle\mathbf{P}|\varphi^{(\mathrm{CM})}\rangle=\tilde{\varphi}(\mathbf{P})$. One gets the (non-normalized) wave function

$$\phi(\mathbf{x})\equiv\langle\mathbf{x}|\mathbb{L}^{(\mathrm{CM})}(\mathbf{y})|\varphi^{(\mathrm{CM})}\rangle=\frac{m}{(2\pi\hbar)^{9/2}}\int\mathrm{d}\mathbf{Q}\mathrm{d}\mathbf{P}\mathcal{F}_r(\mathbf{Q})e^{\frac{i}{\hbar}\mathbf{Q}\cdot(\mathbf{x}-\mathbf{y})}e^{\frac{i}{\hbar}\mathbf{P}\cdot\mathbf{x}}\exp\left(-\frac{r_C^2}{2\hbar^2}\left|(1+k)\mathbf{Q}+2k\frac{\mathbf{P}}{N}\right|^2\right)\tilde{\varphi}(\mathbf{P}),$$

where $\mathcal{F}_r(\mathbf{Q})$ has been defined as

$$\mathcal{F}_r(\mathbf{Q})=\sum_j\exp\left(\frac{i}{\hbar}\sum_{j'}\Lambda_{jj'}\mathbf{Q}\cdot\mathbf{r}_{j'}\right). \tag{15}$$

Now, we use the continuum limit, so that

$$\mathcal{F}_r(\mathbf{Q})=\int\mathrm{d}\mathbf{z}D(\mathbf{z})e^{\frac{i}{\hbar}\mathbf{Q}\cdot\mathbf{z}}, \tag{16}$$

$D(\mathbf{z})$ being the density of particles, and we obtain

$$\phi(\mathbf{x})=\frac{m}{(2\pi\hbar)^{3/2}(\sqrt{2\pi}r_C(1+k)^3)}\int\mathrm{d}\mathbf{z}\mathrm{d}\mathbf{P}D(\mathbf{z})e^{\frac{i}{\hbar}\mathbf{P}\cdot\mathbf{x}}\exp\left(-\frac{|\mathbf{x}-\mathbf{y}+\mathbf{z}|^2}{2r_C^2(1+k)^2}\right)e^{-\frac{2ik}{(1+k)\hbar N}\mathbf{P}\cdot(\mathbf{x}-\mathbf{y}+\mathbf{z})}\tilde{\varphi}(\mathbf{P}).$$

Since

$$\varphi(\mathbf{x}) = \int \mathrm{d}\mathbf{P}\frac{e^{\frac{i}{\hbar}\mathbf{P}\cdot\mathbf{x}}}{(2\pi\hbar)^{3/2}}\tilde{\varphi}(\mathbf{P}),$$

one has

$$\phi(\mathbf{x}) = \frac{m}{(2\pi r_C^2)^{3/2}}\int \mathrm{d}\mathbf{z}D(\mathbf{z})\frac{\exp\left(-\frac{|\mathbf{x}-\mathbf{y}+\mathbf{z}|^2}{2r_C^2(1+k)^2}\right)}{(1+k)^3}\varphi\left(\mathbf{x} - \frac{2k}{(1+k)N}(\mathbf{x}-\mathbf{y}+\mathbf{z})\right). \tag{17}$$

Under the assumption that one can neglect the changes of the macroscopic density $D(\mathbf{y})$ on the length-scale fixed by $r_C(1+k)$, it is clear that the exponential in Eq.(17) varies as a function of $\mathbf{z}$ much faster than the other terms within the integral. By replacing z_i with $z_i + r_C(1+k)$ in the argument of the wave function φ, one obtains a shift of the order of 10^{-36}m, for a macroscopic particle made up of 10^{23} nucleons and a noise temperature of the order of 1K. Hence, we can make the substitution

$$\frac{1}{(2\pi r_C^2(1+k)^2)^{3/2}}\exp\left(-\frac{|\mathbf{x}-\mathbf{y}+\mathbf{z}|^2}{2r_C^2(1+k)^2}\right) \to \delta^3\left(\mathbf{x}-\mathbf{y}+\mathbf{z}\right) \tag{18}$$

in Eq.(17), thus getting

$$\phi(\mathbf{x}) \approx mD(\mathbf{y}-\mathbf{x})\varphi(\mathbf{x}). \tag{19}$$

The Lindblad master equation for the state of the center of mass is given by

$$\frac{\mathrm{d}}{\mathrm{d}t}\hat{\rho}^{(\mathrm{CM})}(t) = -\frac{i}{\hbar}\left[\widehat{H}_{\mathrm{CM}}\,,\,\hat{\rho}^{(\mathrm{CM})}(t)\right] + \frac{\gamma}{m_0^2}\int \mathrm{d}\mathbf{y}\left[\hat{\mathbb{L}}^{(\mathrm{CM})}(\mathbf{y})\hat{\rho}^{(\mathrm{CM})}(t)\hat{\mathbb{L}}^{(\mathrm{CM})\dagger}(\mathbf{y}) - \frac{1}{2}\left\{\hat{\mathbb{L}}^{(\mathrm{CM})\dagger}(\mathbf{y})\hat{\mathbb{L}}^{(\mathrm{CM})}(\mathbf{y}),\hat{\rho}^{(\mathrm{CM})}\right\}\right]. \tag{20}$$

By using Eq.(19) and neglecting the free Hamiltonian contribution, we end up with the equation in the position representation

$$\partial_t\langle\mathbf{x}'|\hat{\rho}^{(\mathrm{CM})}(t)|\mathbf{x}''\rangle = -\Lambda(\mathbf{x}',\mathbf{x}'')\langle\mathbf{x}'|\hat{\rho}^{(\mathrm{CM})}(t)|\mathbf{x}\rangle'', \tag{21}$$

where

$$\Lambda(\mathbf{x}',\mathbf{x}'') \approx \gamma\int \mathrm{d}\mathbf{y}[D^2(\mathbf{y}) - D(\mathbf{y})D(\mathbf{y}+\mathbf{x}'-\mathbf{x}'')]. \tag{22}$$

The same expression was obtained for the original CSL model in [6], under the so-called sharp-scanning approximation. In particular, if we consider a rigid body with constant density D, we get [6]

$$\Lambda(\mathbf{x}',\mathbf{x}'') = \gamma D n_{\mathrm{out}}, \tag{23}$$

with n_{out} the number of particles of the body when its center of mass is in the position $\mathbf{x}'$ that are outside the volume occupied by the body when its center of mass is in $\mathbf{x}''$. Indeed, if n_{out} is equal to the total number of particles (i.e. there is no overlap between the volumes occupied by the macroscopic rigid body when its center of mass is in, respectively, $\mathbf{x}'$ and $\mathbf{x}''$), one recovers Eq.(3) of the main text, up to an irrelevant constant factor $(4\pi)^{3/2}$.

---

[1] Barchielli, A. & Gregoratti, M. *Quantum Trajectories and Measurements in Continuous Time: The Diffusive Case*, Lect. Notes Phys. **782** (Springer, Berlin Heidelberg 2009)

[2] Bassi, A., Lochan, K., Satin, S., Singh, T.P., & Ulbricht, H. Models of wave-function collapse, underlying theories, and experimental tests. *Rev. Mod. Phys.* **85**, 471 (2013)

[3] Adler, S.L. arXiv:1401.0353 (2014)

[4] Arnold, L. *Stochastic Differential Equations: Theory and Applications* (Wiley, New York, 1971)

[5] Bassi, A. & Ghirardi, G.C. Dynamical reduction models. *Phys. Rep.* **379**, 257 (2003)

[6] Ghirardi, G. C., Pearle, P. & Rimini, A. Markov processes in Hilbert space and continuous spontaneous localization of systems of identical particles. *Phys. Rev. A* **42**, 78 (1990).

[7] Vacchini, B. completely positive quantum dissipation. *Phys. Rev. Lett.* **84**, 1374 (2000).

[8] Smirne, A., Vacchini, B. & Bassi, A. Dissipative extension of the Ghirardi-Rimini-Weber model. *Phys. Rev. A* **90**, 062135 (2014)

[9] Vacchini, B. On the precise connection between the GRW master equation and master equations for the description of decoherence. *J. Phys. A: Math. Gen.* **40**, 2463 (2007)

[10] Spohn, H. Approach to equilibrium for completely positive dynamical semigroups of N-level systems. *Rep. Math. Phys.* **10**, 189 (1976); An algebraic condition for the approach to equilibrium of an open N-level system *Lett. Math. Phys.* **2**, 33 (1977)

[11] Vacchini B. & Hornberger, K. Quantum linear Boltzmann equation. *Phys.Rep.* **478**, 71 (2009)
